# Structural Coupling Between Shannon Entropy and Dwell-Time Entropy Across Emotional and Safety-Critical Visual Tasks

**DOI:** 10.3390/jemr19040075

**Published:** 2026-07-09

**Authors:** Yejin Lee, Kwangtae Jung

**Affiliations:** Department of Industrial Design Engineering, Korea University of Technology and Education, Cheonan 31253, Republic of Korea

**Keywords:** eye-tracking, Shannon entropy, dwell-time entropy, visual attention, emotional evaluation, situation awareness

## Abstract

This study investigated the relationship between fixation-frequency-based Shannon entropy and dwell-time-based entropy across two different visual task domains: emotional evaluation of automotive exterior designs and safety-critical monitoring of nuclear power plant emergency scenarios. Although gaze entropy has been widely used to explain emotional responses, task performance, and situation awareness, the relationship between entropy measures derived from fixation counts and fixation durations remains insufficiently examined. Eye-tracking data were analyzed from two experiments with different attentional characteristics. In the emotional visual task, 10 participants evaluated three automotive design images. In the safety-critical task, 20 participants performed four nuclear power plant emergency monitoring scenarios. Shannon entropy and dwell-time entropy were calculated using fixation count and fixation duration distributions across Areas of Interest, respectively. Pearson correlation and simple regression analyses were conducted within each task domain. The results showed strong positive associations between Shannon entropy and dwell-time entropy in both domains. The emotional task showed a correlation of r = 0.844, while the safety-critical task showed a correlation of r = 0.890. These findings suggest that fixation-frequency-based and dwell-time-based entropy measures exhibit substantial overlap across different visual task contexts. However, the observed associations may partly reflect mathematical dependency between fixation frequency and cumulative dwell-time, and the findings should be interpreted as exploratory evidence rather than proof of metric interchangeability. The study highlights that gaze entropy metrics should be interpreted in relation to task-dependent attentional contexts. Higher entropy may be associated with exploratory visual attention in emotional evaluation, whereas lower entropy may be associated with focused monitoring in safety-critical tasks.

## 1. Introduction

This study investigates the structural relationship between fixation-frequency-based (Shannon) gaze entropy and dwell-time-based gaze entropy across emotional and safety-critical visual tasks. While previous research links gaze entropy to behavioral outcomes, the structural alignment between these two metrics—which use different probabilistic bases (fixation counts vs. durations)—remains under-examined.

Eye-tracking has been widely utilized as a representative human factor methodology for analyzing visual attention, cognitive strategies, and information processing characteristics [1]. Since eye movements reflect what information humans attend to and how they explore and interpret their environment, eye-tracking has been regarded as an important objective measure for evaluating cognitive states and behavioral characteristics across various domains [2]. Recently, beyond traditional eye-tracking metrics such as fixation duration and fixation count, gaze entropy has garnered increasing attention as a means to represent the overall dispersion and complexity of gaze behavior using an integrated quantitative index [3,4].

Gaze entropy is a method that quantifies the uncertainty and dispersion of eye movements based on the concept of entropy derived from information theory. In general, gaze entropy is calculated from gaze plots or heatmaps, and representative entropy metrics include Shannon entropy, Markov entropy, dwell-time entropy, and heatmap entropy [5,6]. These gaze entropy measures have been applied in various domains, including medical image interpretation, aviation, sports, webpage evaluation, situation awareness analysis, and emotional design evaluation, to analyze characteristics of human visual exploration [7].

Previous gaze entropy studies have mainly focused on the relationship between entropy and human responses such as task performance, situation awareness, and emotional satisfaction. For example, in emotional design evaluation, designs with higher emotional satisfaction have been reported to produce higher gaze entropy, whereas in emergency operation situations in nuclear power plants, higher levels of situation awareness have been associated with lower gaze entropy [8,9,10,11]. These findings suggest that gaze entropy can serve as an effective indicator for explaining human emotional responses and cognitive states.

However, most previous studies have primarily examined the relationship between specific entropy metrics and human responses, while relatively little attention has been paid to the relationships among different gaze entropy measures calculated using different probabilistic bases. In particular, although both Shannon entropy and dwell-time entropy are derived from Shannon’s information-theoretic entropy formulation, they differ in terms of the attentional mechanisms they may represent. Shannon entropy is calculated based on the relative distribution of fixation counts across Areas of Interest (AOIs), thereby reflecting how frequently users explore various AOIs. In contrast, dwell-time entropy is calculated based on the relative distribution of fixation duration within AOIs, reflecting sustained attention and the depth of information processing associated with specific AOIs.

Therefore, frequent visits to an AOI do not necessarily indicate prolonged attentional engagement with that AOI. For example, users may frequently scan a specific AOI with only brief fixations, whereas another AOI may be visited less often but receive prolonged visual attention. From this perspective, fixation-frequency-based entropy and dwell-time-based entropy may reflect different attentional mechanisms. Consequently, the relationship between Shannon entropy and dwell-time entropy cannot be assumed a priori and requires empirical verification. Nevertheless, few studies have investigated the structural relationship between these two entropy metrics or compared their cognitive meanings across different task domains [12].

In particular, emotional visual exploration and safety-critical visual monitoring involve substantially different cognitive characteristics. In emotional tasks such as automotive exterior design evaluation, users tend to freely explore various design elements to form overall esthetic impressions and emotional preferences, resulting in strong exploratory attention characteristics. In contrast, in safety-critical tasks such as nuclear power plant interface monitoring, users tend to focus on critical information and minimize unnecessary gaze transitions in accordance with goal-directed monitoring principles emphasized in ecological interface design research [13]. Therefore, comparing the relationship between fixation-frequency-based entropy and dwell-time-based entropy across these different task domains may provide important insights into the cognitive meaning of gaze entropy.

Accordingly, this study aims to comparatively analyze the relationship between Shannon entropy and dwell-time entropy in emotional visual tasks and safety-critical visual tasks. To achieve this objective, eye-tracking data obtained from an emotional evaluation experiment involving automotive exterior designs and a situation awareness experiment involving nuclear power plant emergency operation scenarios were analyzed. By examining the correlation between fixation-frequency-based entropy and dwell-time-based entropy across the two domains, this study investigates whether strong associations are consistently observed between the two entropy metrics across different task domains. Furthermore, the study aims to provide theoretical implications regarding the attentional mechanisms reflected by gaze entropy and the task-dependent interpretation of entropy metrics.

To address these objectives, the present study investigated the following research questions:

RQ1. Is there a significant relationship between Shannon entropy and dwell-time entropy in emotional visual tasks?

RQ2. Is there a significant relationship between Shannon entropy and dwell-time entropy in safety-critical visual tasks?

RQ3. Are strong associations between Shannon entropy and dwell-time entropy consistently observed across task domains with different attentional characteristics?

Based on the theoretical distinction between fixation-frequency-based attention and dwell-time-based attention, we hypothesized that the two entropy measures would exhibit a positive relationship across both task domains.

## 2. Theoretical Background

### 2.1. Eye Movements and Visual Attention

Eye movements are one of the representative physiological responses that reflect human visual attention and information processing mechanisms. Humans acquire most information from the external environment through vision, and eye movements indicate which information is selectively attended to during visual understanding processes [14]. Therefore, eye-tracking has been widely utilized as an important human factor methodology for analyzing cognitive states, emotional responses, situation awareness, and decision-making strategies.

In eye-tracking research, fundamental metrics such as fixation duration, fixation count, saccade length, and time to first fixation have been widely used. These basic metrics are effective for analyzing the importance of specific information or Areas of Interest (AOIs). For example, fixation count indicates how frequently a specific AOI is explored, whereas fixation duration represents how long attention is maintained on a particular AOI [1]. However, these conventional eye-tracking metrics primarily reflect isolated characteristics of gaze behavior and therefore have limitations in comprehensively explaining the overall structure of visual exploration and the dispersion characteristics of visual attention.

To overcome these limitations, gaze entropy has recently been introduced as a method for representing the overall dispersion and complexity of gaze behavior using an integrated quantitative index. Gaze entropy quantifies the uncertainty and attentional dispersion of eye movements based on the concept of entropy derived from information theory. A high gaze entropy value indicates that visual attention is broadly distributed across multiple AOIs, whereas a low entropy value indicates that gaze is concentrated on specific AOIs. Due to these characteristics, gaze entropy has been widely utilized as an important analytical measure for explaining human visual exploration strategies and cognitive characteristics.

### 2.2. Shannon Entropy in Eye Movements

Shannon entropy is an entropy calculation method based on the concept of mean information quantity proposed in Shannon’s information theory. In the field of eye-tracking, Shannon entropy has been widely utilized as a quantitative measure for representing how a user’s gaze is distributed across Areas of Interest (AOIs) [15].

A high Shannon entropy value indicates that the user explored multiple AOIs relatively evenly, whereas a low Shannon entropy value indicates that visual attention was concentrated on specific AOIs. Accordingly, Shannon entropy can be utilized to explain visual scanning diversity or gaze exploration tendency.

Previous studies have employed Shannon entropy to analyze various characteristics of human visual exploration behavior. Krejtz et al. [16] analyzed gaze exploration characteristics during artwork viewing using Shannon entropy, while Jungk et al. [17] utilized gaze entropy to evaluate the efficiency of medical monitoring displays. In addition, Chanijani et al. [18] investigated differences in gaze exploration strategies according to learning levels during physics problem-solving tasks using Shannon entropy. These studies demonstrate that Shannon entropy can serve as an effective metric for analyzing human visual exploration behavior and cognitive strategies.

However, because Shannon entropy is calculated solely based on AOI visit frequency, it has a limitation in that it does not reflect how long visual attention remains on a specific AOI. For example, a user may visit a particular AOI very frequently while maintaining only brief fixation durations, whereas another AOI may be visited less frequently but receive prolonged visual attention.

It should be noted that Shannon entropy itself is a general information-theoretic measure of uncertainty. In the present study, Shannon entropy was operationalized using fixation count distributions across AOIs, whereas dwell-time entropy was calculated using dwell-time distributions. Thus, the two metrics share the same mathematical formulation but differ in their probabilistic basis.

Therefore, although Shannon entropy can reflect the frequency-based characteristics of visual attention, it may not fully explain the depth of attentional engagement. From this perspective, dwell-time entropy, which reflects the duration of attention allocated to AOIs, can be utilized as an alternative metric for explaining gaze behavior.

### 2.3. Dwell-Time Entropy in Eye Movements

Dwell-time entropy is an entropy metric proposed to reflect the duration of gaze allocation within Areas of Interest (AOIs) [3,4]. A high dwell-time entropy value indicates that visual attention is distributed relatively evenly across multiple AOIs for comparable durations of time, whereas a low dwell-time entropy value indicates prolonged concentration on specific AOIs. Accordingly, dwell-time entropy can be interpreted as a metric reflecting sustained attention or characteristics of cognitive engagement.

In particular, dwell-time entropy is meaningful because it reflects processing duration rather than merely the frequency of gaze visits. In human visual information processing, prolonged fixation on specific information may indicate deep cognitive processing or high attentional engagement. Therefore, dwell-time entropy may reflect attentional mechanisms different from those represented by Shannon entropy.

For example, even if a user explores a particular AOI very frequently, Shannon entropy may become high, while dwell-time entropy remains relatively low if each fixation duration is brief. Conversely, an AOI may be visited less frequently but receive prolonged fixations, resulting in relatively high dwell-time entropy. Therefore, fixation-frequency-based attention and dwell-time-based attention may represent different characteristics of visual attention.

### 2.4. Gaze Entropy Across Emotional and Safety-Critical Tasks

Gaze entropy may have different cognitive meanings depending on the task domain. In particular, emotional visual exploration and safety-critical visual monitoring differ substantially in that they require distinct attentional mechanisms.

In emotional visual tasks, users tend to freely explore various design elements in order to form esthetic impressions and emotional preferences regarding a product or design, resulting in strong exploratory attention characteristics. In emotional evaluations of automotive exterior designs, previous studies have reported that designs with higher emotional satisfaction tend to produce higher gaze entropy. This finding can be interpreted as indicating that users actively explore multiple design elements rather than focusing only on specific components.

In contrast, safety-critical tasks such as nuclear power plant interface monitoring involve goal-directed attention characteristics, in which users selectively focus on critical information in order to maintain situation awareness. In emergency operation situations in nuclear power plants, higher levels of situation awareness have been associated with lower Shannon entropy and dwell-time entropy. This tendency may indicate that users with higher situation awareness reduce unnecessary gaze transitions by concentrating their visual attention on important AOIs.

Although emotional visual tasks and safety-critical visual tasks require different attentional strategies, based on the theoretical distinction between fixation-frequency-based attention and dwell-time-based attention, a positive relationship between the two entropy measures was expected across task domains. Although emotional visual tasks and safety-critical visual tasks require different attentional strategies, a positive relationship between fixation-frequency-based entropy and dwell-time-based entropy may be expected because both metrics are derived from visual attention allocation processes. Therefore, examining whether this relationship remains stable across different task domains may provide important theoretical insights into the attentional meaning of gaze entropy.

Accordingly, this study aims to comparatively analyze the relationship between Shannon entropy and dwell-time entropy across different task domains in order to investigate what types of attentional mechanisms are reflected by fixation-frequency-based attention and dwell-time-based attention, and how the cognitive meaning of this relationship varies according to task characteristics.

## 3. Method

### 3.1. Overview of the Study

This study was conducted to comparatively analyze the relationship between fixation-frequency-based gaze entropy and dwell-time-based gaze entropy in emotional visual tasks and safety-critical visual tasks. To achieve this objective, two eye-tracking experimental datasets with different cognitive characteristics were utilized. The first experiment involved an emotional satisfaction evaluation of automotive exterior designs, while the second experiment involved a situation awareness assessment during emergency operation scenarios in a nuclear power plant. In both experiments, Shannon entropy and dwell-time entropy were calculated based on eye-tracking data, and the relationships between the two entropy metrics were comparatively analyzed within each domain.

The primary objective of this study was to investigate whether strong associations are consistently observed between fixation-frequency-based entropy and dwell-time entropy across different task domains.

### 3.2. Experiment 1: Emotional Visual Task

#### 3.2.1. Participants

Ten students majoring in design at K University (5 males and 5 females) participated in the emotional domain experiment. The mean age of the participants was 24.3 years (SD = 1.1), and all participants had normal vision.

#### 3.2.2. Experimental Stimuli

**Figure 1:**
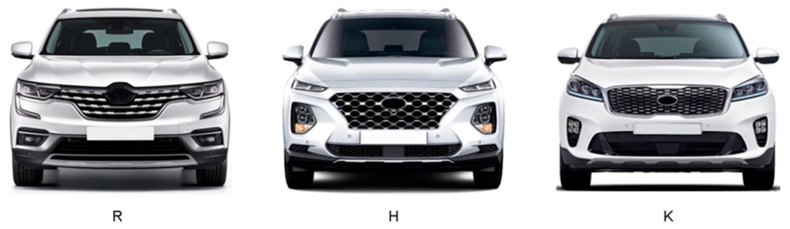
SUV images for the experiment.

Three front-view SUV design images produced by representative automobile manufacturers in Korea were used as experimental stimuli for the emotional evaluation task (Figure 1). Since the front exterior of an automobile plays a critical role in forming consumers’ emotional impressions, front-view vehicle images were selected as the experimental stimuli. To eliminate the influence of brand image, automobile logos were removed from all images. In addition, to minimize the influence of color, all vehicle images were standardized to the same white color.

#### 3.2.3. Experimental Procedure

**Figure 2:**
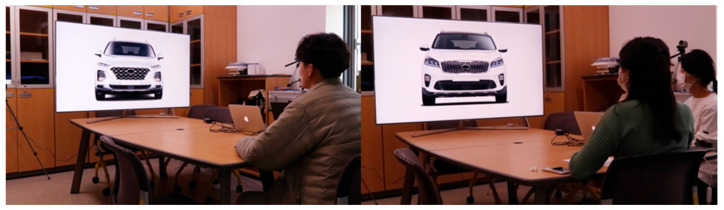
Experimental scene showing a participant wearing Tobii Pro Glasses 2 during the emotional evaluation task.

The experiment was conducted in a quiet laboratory environment using Tobii Pro Glasses 2 (Tobii AB, Stockholm, Sweden) as the eye-tracking device (Figure 2). Participants were seated comfortably in front of a 65-inch display and observed each automobile image for 20 s. The distance between the participants and the display was maintained at 2 m. After viewing each image, participants evaluated their emotional satisfaction with the automobile design using a 5-point Likert scale. To minimize order effects, the presentation order of the automobile images was randomized across participants.

The eye-tracking system used in this study was Tobii Pro Glasses 2, a wearable device consisting of a head unit, a recording unit, and controller software. The gaze sampling frequency was 100 Hz, and corneal reflection with dark pupil tracking was employed. The average binocular accuracy under optimal conditions (illumination = 300 lux, distance = 1.5 m, gaze angle < 15°) was 0.62° (SD = 0.23), and precision was 0.05° (SD = 0.10), computed as the root mean square (RMS) of successive gaze points. The detected gaze ratio was 99% (SD = 1.7). Calibration was conducted individually for each participant by having them fixate on the center of a calibration card positioned at 0.75–1.25 m, and the calibration quality was verified via the controller software.

#### 3.2.4. AOI Definition

**Figure 3:**
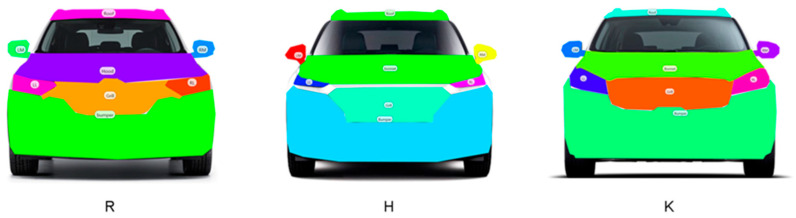
Areas of Interest (AOIs) defined for the automotive exterior design evaluation task. Eight AOIs corresponding to major vehicle design components were identified and used for entropy calculations.

To calculate gaze-plot-based entropy, the front exterior design of the automobile was divided into AOIs according to major design elements. The AOIs consisted of eight regions: Bumper, Grill, Hood, Roof, Left Light, Right Light, Left Mirror, and Right Mirror (Figure 3). Shannon entropy and dwell-time entropy were calculated based on the fixation count and fixation duration data obtained for each AOI [10]. Because the objective of the present study was to examine the relationship between Shannon entropy and dwell-time entropy within the same task domain, both entropy measures were calculated using identical AOI definitions within the emotional task. Therefore, any influence of AOI segmentation affected both entropy measures equally within this task domain.

Entropy values were calculated separately for each participant–image combination. Thus, the statistical analyses were conducted using 30 participant–image observations (10 participants × 3 vehicle images). Each observation represented a unique combination of one participant and one stimulus image.

### 3.3. Experiment 2: Safety-Critical Visual Task

#### 3.3.1. Participants

Twenty university students without professional knowledge related to nuclear power plants participated in the nuclear domain experiment (10 males and 10 females). The average age of the participants was 24.0 years (SD = 1.72). The participants were divided into a trained group and an untrained group. The trained group consisted of participants who had received sufficient prior training regarding nuclear power plant accident scenarios, whereas the untrained group received only basic explanations about the accident situations.

#### 3.3.2. Experimental Task

As safety-critical visual tasks, four representative emergency accident scenarios in nuclear power plants were employed: LOCA (Loss of Coolant Accident), SGTR (Steam Generator Tube Rupture), SLB (Steam Line Break), and LOV (Loss of Voltage). Participants performed a task in which they observed changes in nuclear power plant state variables displayed on the screen and identified the corresponding accident situation.

To identify the accident scenarios, six major indicators were utilized: reactor power (Rx Power), reactor vessel water level (Rv Water Level), pressurizer pressure (PZR Pressure), containment radiation (CTMT Radiation), containment pressure (CTMT Pressure), and secondary-side radiation (Sec. Radiation) (Table 1). Each accident type was distinguished according to the characteristic change patterns of these variables.

#### 3.3.3. Experimental Procedure

**Figure 4:**
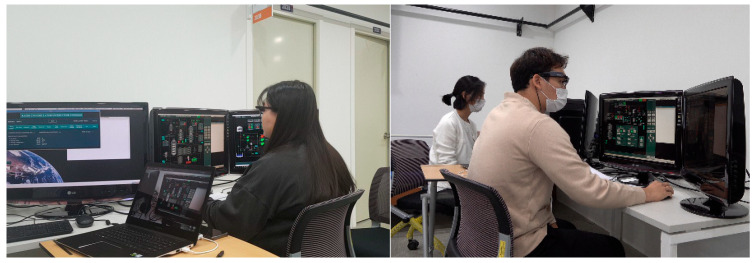
Experimental scene showing participants during the situation-awareness task using a nuclear power plant simulator.

The experiment was conducted in a quiet laboratory environment. Participants observed the nuclear power plant emergency simulator interface while wearing Tobii Glasses 2 eye-tracking equipment (Figure 4). The experimental interface consisted of three display screens representing the reactor cooling system, the main steam system, and the residual heat removal system. Participants observed changes in the state variables for approximately 2 min and then identified the current accident situation. After completing the experiment, participants completed the SART (Situation Awareness Rating Technique) questionnaire [19] to evaluate their level of situation awareness.

#### 3.3.4. AOI Definition

**Figure 5:**
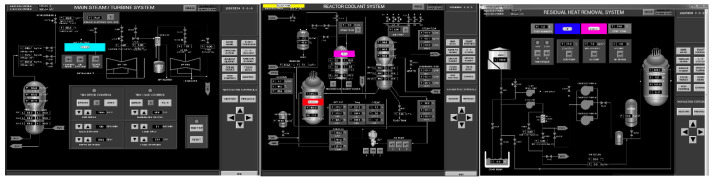
Areas of Interest (AOIs) defined for the nuclear power plant situation-awareness task. AOIs corresponded to key interface indicators used for accident diagnosis and monitoring.

The AOIs in the nuclear power plant interface were defined as the major indicator regions required for accident identification. Each state-variable display area was designated as an AOI. Shannon entropy and dwell-time entropy were calculated using the fixation count and fixation duration data obtained from each AOI. The AOIs were defined according to the information elements required for accident identification [20,21] (Figure 5). Both entropy measures were calculated using the same AOI structure within the safety-critical task, allowing the relationship between the two entropy metrics to be examined under identical segmentation conditions.

Entropy values were calculated separately for each participant–scenario combination. Thus, the statistical analyses were conducted using 79 participant–scenario observations (20 participants × 4 accident scenarios). Each observation represented a unique combination of one participant and one accident scenario.

### 3.4. Calculation of Gaze Entropy

The general formula for spatial gaze entropy is as follows [15,21]:

$$H=-\overset{n}{\underset{i=1}{\sum }}P_{i}log_{2}P_{i}$$

where P_i_ represents the relative probability assigned to the *i*-th AOI. For Shannon entropy, P_i_ was defined as the proportion of fixation counts in the *i*-th AOI relative to the total number of fixations across all AOIs. For dwell-time entropy, P_i_ was defined as the proportion of total dwell-time spent in the *i*-th AOI relative to the total dwell-time across all AOIs. Thus, the two entropy measures share the same mathematical formulation but differ in the probabilistic basis used for calculation.

Although both entropy metrics employ the same mathematical structure derived from Shannon’s information theory, they differ in the way the probability distribution is defined. Specifically, Shannon entropy reflects the frequency of AOI visits, whereas dwell-time entropy reflects the distribution of fixation duration across AOIs.

### 3.5. Statistical Analysis

Pearson correlation analysis was conducted to examine the relationship between Shannon entropy and dwell-time entropy in both the emotional and safety-critical task domains. In addition, simple linear regression analyses were also performed to investigate the extent to which fixation-frequency-based entropy was associated with dwell-time-based entropy.

The purpose of the cross-domain analysis was not to statistically compare the magnitude of the correlation coefficients, but to examine whether strong associations between Shannon entropy and dwell-time entropy were consistently observed across different task domains. This analysis was conducted to examine the stability of the entropy relationship across different attentional contexts.

The entropy values were not normalized by maximum possible entropy because the purpose of the present study was not to compare absolute entropy magnitudes across task domains. This decision was guided by the specific research objective of examining the relationship between two entropy metrics within each task domain rather than comparing entropy magnitudes across domains, consistent with recent recommendations emphasizing alignment between research questions and eye-tracking operationalization choices [22]. Instead, the analyses focused on examining the relationship between Shannon entropy and dwell-time entropy within each task domain.

Because the study focused on exploring the relationship between two entropy metrics across multiple task instances, participant–image and participant–scenario observations were used as the unit of analysis.

Because multiple observations were obtained from the same participants, the observations were not fully independent. Therefore, the results should be interpreted with caution, and the present analyses are intended as an exploratory examination of the relationship between the two entropy metrics.

To further address the repeated-measures issue, an additional sensitivity analysis was conducted using participant-level aggregated data. For the emotional visual task, Shannon entropy and dwell-time entropy were averaged across the three automotive images for each participant. For the safety-critical visual task, Shannon entropy and dwell-time entropy were averaged across the four accident scenarios for each participant. Pearson correlation analyses were then repeated using these participant-level mean values to examine whether the observed associations remained robust after removing within-participant repeated observations.

## 4. Results

### 4.1. Relationship Between Shannon Entropy and Dwell-Time Entropy in Emotional Visual Tasks

**Figure 6:**
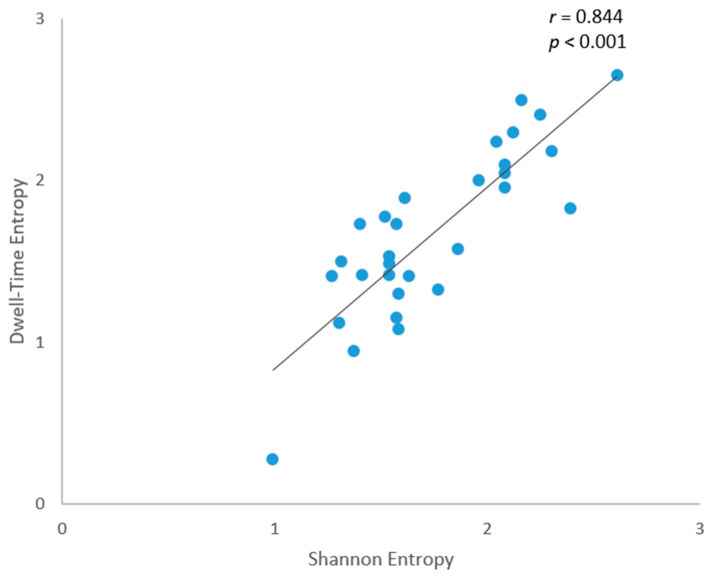
Scatterplot showing the relationship between Shannon entropy and dwell-time entropy in the emotional visual task. Each point represents one participant-image observation (*n* = 30), and the solid line represents the fitted linear regression line.

The relationship between Shannon entropy and dwell-time entropy was analyzed in the emotional visual task involving automotive exterior design evaluation. The results revealed a strong positive correlation between the two entropy metrics (Figure 6). Pearson correlation analysis indicated that Shannon entropy was strongly associated with dwell-time entropy (r = 0.844, *p* < 0.001), suggesting that fixation-frequency-based attention distribution and dwell-time-based attentional engagement are closely related during emotional visual exploration.

In addition, linear regression analysis demonstrated that Shannon entropy was significantly associated with dwell-time entropy in the emotional visual task (R^2^ = 0.710, F(1,28) = 69.54, *p* < 0.001). The regression model indicated that increases in fixation-frequency-based entropy were associated with corresponding increases in dwell-time-based entropy.

### 4.2. Relationship Between Shannon Entropy and Dwell-Time Entropy in Safety-Critical Visual Tasks

**Figure 7:**
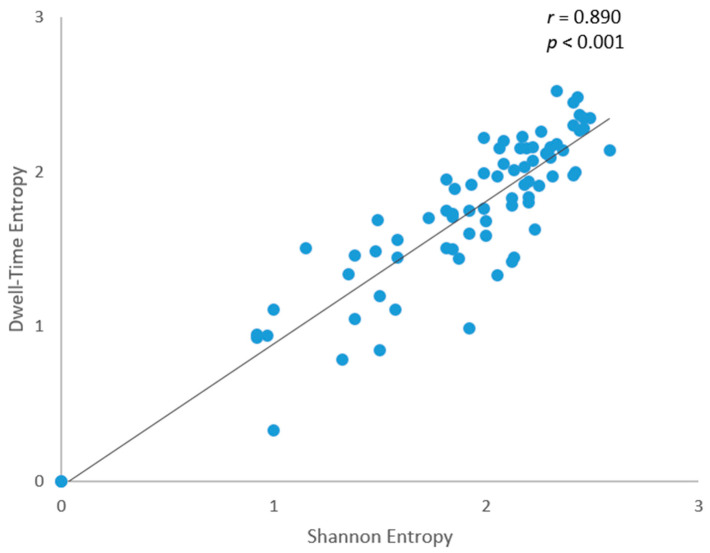
Scatterplot showing the relationship between Shannon entropy and dwell-time entropy in the safety-critical visual task. Each point represents one participant-scenario observation (*n* = 79), and the solid line represents the fitted linear regression line.

The relationship between Shannon entropy and dwell-time entropy was also analyzed in the safety-critical visual task involving nuclear power plant interfaces (Figure 7). The results revealed a strong positive correlation between the two entropy metrics. Pearson correlation analysis indicated that Shannon entropy was strongly associated with dwell-time entropy (r = 0.890, *p* < 0.001), demonstrating a stable relationship between fixation-frequency-based attention distribution and dwell-time-based attentional engagement even in a safety-critical monitoring environment.

Linear regression analysis further confirmed the strong relationship between Shannon entropy and dwell-time entropy in the safety-critical visual task (R^2^ = 0.792, F(1,77) = 294.777, *p* < 0.001). These findings provide additional evidence of a strong association between fixation-frequency-based entropy and dwell-time-based entropy in nuclear power plant monitoring tasks, although part of this association may reflect the inherent dependency between fixation frequency and cumulative dwell-time.

### 4.3. Cross-Domain Consistency of Entropy Relationships

A comparative analysis of entropy relationships across emotional visual tasks and safety-critical visual tasks revealed consistently strong positive correlations between Shannon entropy and dwell-time entropy in both task domains despite their substantially different cognitive characteristics. In the emotional domain, the correlation coefficient between the two entropy metrics was r = 0.844, whereas the nuclear power plant domain showed a correlation coefficient of r = 0.890. Both correlations were statistically highly significant (*p* < 0.001).

These findings suggest that strong associations between fixation-frequency-based entropy and dwell-time entropy were observed across different task domains. In other words, AOIs that were explored more frequently also tended to receive relatively prolonged visual attention regardless of differences in task characteristics. Although the cognitive demands of emotional visual exploration and safety-critical monitoring differ substantially, both domains demonstrated similarly strong relationships between Shannon entropy and dwell-time entropy. Nevertheless, because only two task domains and relatively small participant samples were examined, these findings should be interpreted as preliminary evidence rather than definitive proof of a stable cross-domain relationship. Because repeated observations from the same participants were included in the primary analyses, the statistical significance of the original correlations should be interpreted with caution. Therefore, an additional participant-level aggregation analysis was conducted as a sensitivity analysis to examine whether the observed associations remained robust after removing within-participant repeated observations. The sensitivity analysis yielded correlation coefficients comparable to those obtained from the original analyses, supporting the robustness of the observed relationships.

### 4.4. Sensitivity Analysis Using Participant-Level Aggregation

To examine whether the observed associations remained robust after addressing the repeated-measures structure of the data, an additional sensitivity analysis using participant-level aggregation was conducted (Table 2).

For the emotional visual task, entropy values were averaged across the three automotive images for each participant. The participant-level analysis revealed a strong positive correlation between Shannon entropy and dwell-time entropy (r = 0.882, *p* = 0.001, N = 10). Similarly, for the safety-critical visual task, entropy values were averaged across the four accident scenarios for each participant. A strong positive correlation was also observed between Shannon entropy and dwell-time entropy (r = 0.912, *p* < 0.001, N = 20). The participant-level aggregated analyses yielded correlation coefficients comparable to those obtained from the original analyses, indicating that the observed relationships were not driven solely by within-participant repeated observations.

These results indicate that the strong positive associations between Shannon entropy and dwell-time entropy were maintained even after removing within-participant repeated observations. Therefore, the participant-level aggregation sensitivity analysis provides additional evidence that the primary findings were robust despite the repeated-measures structure of the original dataset. Accordingly, the interpretation of the observed associations does not rely solely on the original participant–image or participant–scenario analyses but is additionally supported by participant-level aggregated analyses.

### 4.5. Relationship Between Frequency-Based and Dwell-Time-Based Entropy

The most important finding of this study is that strong associations between Shannon entropy and dwell-time entropy were consistently observed across different visual task environments. Although emotional visual exploration and safety-critical monitoring require different attentional mechanisms, both domains demonstrated highly stable correlations between Shannon entropy and dwell-time entropy (r > 0.87).

These findings suggest that a structural relationship may exist between AOI visit frequency and attentional engagement duration during human visual attention allocation processes. In other words, AOIs that are explored more frequently may also tend to receive relatively prolonged attentional engagement.

However, the present findings do not necessarily indicate that fixation-frequency-based attention and dwell-time-based attention represent identical attentional mechanisms. In the emotional domain, entropy was associated with exploratory visual attention characteristics, whereas in the safety-critical domain, entropy reflected focused attention and efficient monitoring behavior. These results suggest that, although the structural relationship between Shannon entropy and dwell-time entropy remains stable across task domains, the cognitive interpretation of entropy may vary according to task characteristics.

Therefore, the relationship between Shannon entropy and dwell-time entropy may reflect not only mathematical similarity derived from Shannon’s information theory, but also structural attentional coupling occurring during human visual attention allocation processes.

## 5. Discussion

### 5.1. Structural Relationship Between Shannon Entropy and Dwell-Time Entropy

This study comparatively analyzed the relationship between Shannon entropy and dwell-time entropy across emotional visual tasks and safety-critical visual tasks. The results revealed consistently strong positive correlations between the two entropy metrics, irrespective of the substantially different task domains. In the automotive exterior design evaluation task, the correlation coefficient between Shannon entropy and dwell-time entropy was r = 0.844, whereas the nuclear power plant interface task showed a correlation coefficient of r = 0.890. Importantly, the participant-level aggregation analysis showed that these positive associations remained strong after removing within-participant repeated observations. This finding suggests that the observed relationships were not solely attributable to the repeated-measures structure of the original dataset.

These findings suggest that the relationship between Shannon entropy and dwell-time entropy was consistently observed across substantially different task domains. Because the analyses focused on within-domain relationships rather than direct comparisons of entropy magnitude across domains, differences in AOI structure are unlikely to fully account for the observed associations. Moreover, both entropy measures within each task domain were calculated using identical AOI definitions. Therefore, differences in AOI segmentation are unlikely to fully explain the observed strong associations between Shannon entropy and dwell-time entropy. However, the extent to which this relationship reflects general attentional characteristics, mathematical dependency, or both remains an important topic for future research. Previous eye-tracking studies have generally treated fixation count and fixation duration as independent attention metrics. However, the present findings indicate that AOIs explored more frequently also tend to receive relatively prolonged attentional engagement.

In particular, this study is meaningful in that such robust strong associations were consistently observed across substantially different cognitive environments, including emotional visual exploration and safety-critical monitoring. These findings suggest that the relationship between Shannon entropy and dwell-time entropy may not be limited to specific task characteristics. However, given the relatively small sample sizes and the limited number of task domains examined, the present findings should be regarded as preliminary evidence requiring further replication before broader generalizations can be made.

A plausible explanation is that fixation frequency and fixation duration do not constitute entirely independent dimensions of visual attention. AOIs that attract repeated visits are also likely to receive longer cumulative processing time, resulting in similar probability distributions and consequently similar entropy values. This observation suggests that the two entropy metrics may capture partially overlapping aspects of visual attention rather than entirely independent attentional constructs.

An alternative explanation for the strong correlations observed in the present study is the inherent dependency between fixation frequency and cumulative dwell-time. Because total dwell-time is partly composed of repeated fixations, AOIs receiving more fixations are also likely to accumulate longer viewing durations. Consequently, a substantial relationship between fixation count-based entropy and dwell-time-based entropy may be expected to some extent. Therefore, the observed correlations may reflect both mathematical dependency arising from the underlying eye-tracking measures and attentional allocation processes. Future studies should further investigate the relative contributions of these factors using additional analytical approaches.

The present findings do not imply that the observed associations arise solely from attentional mechanisms. Rather, they demonstrate that strong relationships between Shannon entropy and dwell-time entropy were consistently observed across two substantially different task domains despite their different probabilistic definitions. Although the two entropy measures converged strongly in the present analyses, the results do not establish that they are functionally equivalent or interchangeable. Future studies employing agreement analyses, equivalence testing, or predictive-validity comparisons are required to address these questions directly.

### 5.2. Task-Dependent Interpretation of Gaze Entropy

Although the structural relationship between Shannon entropy and dwell-time entropy was consistently observed across both task domains, the cognitive meaning of entropy differed according to task characteristics.

In emotional visual tasks, higher entropy was associated with visual attention patterns consistent with exploratory viewing and attentional dispersion across multiple design elements. Participants tended to repeatedly explore various design components rather than concentrating attention on specific AOIs in order to form overall esthetic impressions and emotional preferences. These findings may suggest that broad visual exploration strategies are involved in aesthetic evaluation processes; however, the present study did not directly measure emotional preference or aesthetic judgment outcomes.

In contrast, lower entropy in safety-critical visual tasks was associated with focused attention and efficient monitoring behavior. Participants appeared to concentrate visual attention on critical AOIs required for accident identification while minimizing unnecessary gaze transitions. These findings are consistent with the interpretation that focused attention and goal-directed monitoring strategies may be important characteristics of safety-critical environments. These findings are consistent with previous vigilance-task studies suggesting that efficient monitoring behavior is associated with selective visual attention and reduced unnecessary gaze transitions [23].

Therefore, even when the same gaze entropy metric is applied, different cognitive interpretations may be required depending on the task domain. Because the present study primarily examined the relationship between two entropy metrics rather than direct behavioral outcomes, these interpretations should be regarded as theoretically informed explanations rather than direct empirical evidence. High entropy does not necessarily indicate an efficient attentional state, and the meaning of entropy should be interpreted in consideration of task demands and attentional context.

### 5.3. Theoretical and Practical Implications

This study suggests that the definition of probability distribution plays an important role in the interpretation of gaze entropy metrics. Although Shannon entropy and dwell-time entropy share the same mathematical structure derived from Shannon’s information theory, they differ in that they are calculated based on different attentional variables, namely fixation count and fixation duration. Because both metrics are derived from the same entropy formulation and are calculated from the same eye-tracking dataset, part of the observed association may be expected. Nevertheless, the consistency of these associations across different task domains provides useful empirical evidence regarding the practical relationship between fixation-frequency-based and dwell-time-based entropy measures.

Overall, the present findings consistently revealed strong correlations between the two entropy metrics across different task domains. These results suggest that fixation-frequency-based entropy and dwell-time-based entropy share substantial overlap despite being derived from different attentional variables. At the same time, the attentional meaning of entropy was shown to vary according to task characteristics, indicating the necessity of a task-dependent interpretation framework for gaze entropy analysis.

The findings of this study may provide a theoretical foundation for gaze entropy-based attentional analysis in various fields, including human factors, cognitive ergonomics, UX evaluation, emotional design assessment, and safety-critical interface analysis.

### 5.4. Limitations and Future Research

This study has several limitations. Although strong associations were consistently observed between Shannon entropy and dwell-time entropy, the findings should be interpreted in light of several methodological considerations, including the repeated-measures structure of the data, relatively small sample sizes, differences in AOI definitions, and the potential mathematical dependency between the two entropy measures. These issues are discussed below and should be addressed more rigorously in future research.

First, the present study interpreted attentional mechanisms solely based on eye-tracking data. Future work should employ multimodal physiological analyses incorporating EEG, GSR, and HRV to investigate entropy-based attentional mechanisms more deeply.

Second, the analysis was conducted using only two task domains, which may limit the generalizability of the findings. Furthermore, the sample sizes were relatively small, and the emotional task involved design-major students only. Although the primary purpose of this study was not to establish population estimates but to examine the structural relationship between the two entropy metrics, replication using larger and more diverse participant samples is recommended.

Furthermore, the observed associations across two different task domains suggest the possibility of a broader relationship between fixation-frequency-based and dwell-time-based entropy measures; further studies using larger samples, additional task domains, and multimodal physiological measures are required before broader generalization can be made.

To further examine the influence of the repeated-measures structure, an additional participant-level aggregation sensitivity analysis was conducted. The results showed that the associations between Shannon entropy and dwell-time entropy remained strong in both the emotional task and the safety-critical task after removing within-participant repeated observations. These findings suggest that the main conclusions are robust despite the repeated-measures structure of the original dataset. Nevertheless, participant-level aggregation does not fully account for all sources of dependency or random variation. Therefore, future studies should employ mixed-effects models to more comprehensively model participant-level variability as well as stimulus- and scenario-level random effects [24]. The participant-level aggregation sensitivity analysis demonstrated that the correlation coefficients remained comparable to those obtained from the original analyses, indicating that the observed relationships were not solely attributable to the repeated-measures structure of the data. Accordingly, although the *p*-values obtained from the original analyses should be interpreted with appropriate caution because of the repeated observations, the consistency of the aggregated analyses provides additional support for the robustness of the reported associations.

In addition, the emotional and safety-critical tasks employed different AOI structures. Because entropy values were not normalized by maximum possible entropy, the absolute magnitude of entropy values may be influenced by AOI definition and segmentation. However, the primary objective of the present study was not to compare entropy magnitudes across task domains but to examine the relationship between Shannon entropy and dwell-time entropy within each task domain. Future studies may investigate whether similar findings are obtained using normalized entropy measures.

Finally, the present study did not directly distinguish between mathematical dependency arising from entropy construction and cognitive mechanisms underlying visual attention. Future studies should employ alternative entropy formulations, simulation-based approaches, or independent behavioral measures to further disentangle these effects. For example, transition-based entropy measures that incorporate sequential gaze movements between AOIs may provide additional insights into attentional allocation beyond fixation-frequency-based and dwell-time-based entropy measures [25].

In addition, task-specific interpretations of entropy were based on theoretical considerations and the previous literature rather than direct analyses linking entropy values to behavioral outcomes, emotional ratings, task performance, or situation awareness measures. Because entropy measures are affected by AOI number, size, and segmentation strategy, differences in AOI definition may partially influence entropy values and their interpretation [26]. Future studies should investigate these relationships more directly.

## 6. Conclusions

This study comparatively investigated the relationship between fixation-frequency-based gaze entropy and dwell-time-based gaze entropy across emotional visual tasks and safety-critical visual tasks. Eye-tracking data obtained from automotive exterior design evaluation tasks and nuclear power plant situation awareness tasks were analyzed to examine the relationship between Shannon entropy and dwell-time entropy.

The results demonstrated strong and consistent associations between fixation-frequency-based entropy and dwell-time-based entropy across both task domains. Additional participant-level aggregation analyses further confirmed that these associations remained strong after removing within-participant repeated observations. These associations may reflect shared characteristics of attentional allocation as well as the inherent dependency between fixation frequency and cumulative dwell-time.

However, the cognitive meaning of entropy differed according to task domain. In emotional visual tasks, higher entropy was associated with exploratory attention characteristics, whereas in safety-critical visual tasks, lower entropy was associated with focused attention and efficient monitoring behavior. These findings indicate that gaze entropy metrics should be interpreted not only in terms of entropy magnitude itself, but also in consideration of task-dependent attentional contexts.

Therefore, this study provides exploratory evidence that fixation-frequency-based entropy and dwell-time-based entropy exhibit strong and consistent associations across different task domains, while their cognitive interpretation remains dependent on task-specific attentional contexts. These findings provide preliminary theoretical implications for gaze entropy-based attentional analysis. Further validation using larger and more diverse participant samples and additional task domains is required to establish the generalizability of the observed relationships.

## Figures and Tables

**Table 1:** Changes in six indicators in four accident situations (↑: increase, ↓: decrease, —: no significant change).

	Rx Power	Rv Water Level	PZR Pr.	CTMT Rad	CTMT Pr.	Sec. Rad
LOCA	↓	↓	↓	↑	↑	―
SGTR	↓	↓	↓	―	―	↑
SLB	↓	―	↓	―	↑	―
LOV	↓	―	↓	―	―	―

**Table 2:** Comparison of the original analyses and participant-level aggregation sensitivity analyses.

Task Domain	Original Analysis	Participant-Level Aggregation
Emotional visual task	r = 0.844 (N = 30, *p* < 0.001)	r = 0.882 (N = 10, *p* < 0.001)
Safety-critical visual task	r = 0.890 (N = 79, *p* < 0.001)	r = 0.912 (N = 20, *p* < 0.001)

Note: For the safety-critical task, the original analysis included 79 participant–scenario observations because one participant–scenario observation was missing. Participant-level aggregation retained all 20 participants because each participant contributed valid data from the remaining scenarios.

## Data Availability

The raw data supporting the conclusions of this article will be made available by the authors on request.
